# Advantages of Continuous-Valued Risk Scores for Predicting Long-Term Costs: The Framingham Coronary Heart Disease 10-Year Risk Score

**DOI:** 10.20900/agmr20190004

**Published:** 2019-06-06

**Authors:** Sarah Zheng, Benjamin Lubin, Rhoda Au, Joanne M. Murabito, Emelia J. Benjamin, Michael Shwartz

**Affiliations:** 1 University of Victoria Gustavson School of Business, 3800 Finnerty Rd., Victoria, BC, V8P 5C2, Canada; 2 Information Systems, Boston University Questrom School of Business, 595 Commonwealth Avenue, Boston, MA 02215, USA; 3 Department of Anatomy & Neurobiology and Neurology, Boston University School of Medicine, Boston, MA 02118, USA; 4 Department of Epidemiology, Boston University School of Public Health, Boston, MA 02118, USA; 5 Framingham Heart Study, 73 Mount Mayte Ave., suite 2, Framingham, MA 01702, USA; 6 Department of Medicine, Boston University School of Medicine, Boston, MA 02118, USA; 7 Operations and Technology Management Department, Boston University, Questrom School of Business, Boston, MA 02215, USA

**Keywords:** risk scores, Framingham risk score, Medicare costs, cardiovascular disease

## Abstract

**Background:**

The few studies that have examined the relationship between midlife cardiovascular disease risk and longer-term costs have differentiated risk using a small number of risk categories. In this paper, we illustrate the advantages of a continuous-valued score to examine the relationship between risk and longer-term costs: the Framingham 10-year coronary heart disease risk score.

**Methods:**

Our study cohort consisted of 1333 Second Generation Framingham Heart Study participants enrolled in fee-for-service Medicare for at least 8 quarters and who had a risk score assessment between age 40 and 50 years. We used generalized linear models to examine the relationships between quarterly Medicare costs and risk scores.

**Results:**

Using risk categories defined by the Framingham score, the cost differences between a low and high risk group were 40% to over 200% greater than differences in comparable studies using a small number of risk categories. A continuous-valued score facilitates comparison of the cost consequences of impacting risk score changes. For example, an intervention that is able to reduce a person’s score change between midlife and later-life from the 75th percentile to the 25th percentile would result in almost a 20% reduction in longer-term costs. In contrast, an intervention that is able to reduce a person’s midlife score from the 75th percentile to the 25th percentile would result in a 38% reduction in costs.

**Conclusions:**

A continuous-valued risk score has advantages compared to defining risk based on a small number of risk categories.

## BACKGROUND

The American Heart Association reports that 92.1 million adults (>1 in 3) have cardiovascular disease (CVD)[1]. Total costs for CVD, which include both directs costs (about 68% of the total) and indirect costs (due to lost productivity from premature mortality) and which in 2008 accounted for about 27% of Medicare expenditures [2], are projected to increase from $579 billion in 2012 to $1208 billion in 2030 [1].

Despite the importance of CVD, very few papers have examined the economic implications of CVD risk profiles [3–8] and none, to our knowledge, the economic implications of changes in profiles. Only three studies have examined the association between risk profiles and costs that occur many years after risk assessment. Daviglus *et al.* analyzed costs that occurred over an 11-year period beginning about 13 years on average after risk assessment [3]; Willis *et al.* examined costs that occurred up to 23 years after risk assessment [4]; and Allen *et al.*, expanding and extending analysis of the Daviglus *et al.* cohort, analyzed average annual costs that occurred on average beginning 14 years after risk assessment and extended over a 26-year period [5]. Understanding the long-term cost implications of CVD risk profiles adds an economic dimension to the already well-known association of CVD risk profile with morbidity and mortality.

The Framingham Heart Study (FHS) has been invaluable in identifying risk factors for CVD and has contributed to a wide range of interventions to reduce risk and improve population health [9]. Recently, the FHS obtained from the Centers for Medicare and Medicaid Services (CMS) Medicare cost data on the Framingham cohorts. Using these data, we examined the relationship between Medicare costs and the Framingham CHD 10-year risk prediction score [10,11], assessed at midlife and at later-life. We analyzed costs that, on average, occurred over almost a 7-year period that began over 23 years after risk score assessment.

Daviglus *et al.* [3], Willis *et al.* [4] and Allen *et al.* [5] grouped study participants into a small number of CVD risk categories based on levels of risk factors. In contrast, the Framingham score is a continuous-valued numerical score. In what follows, we illustrate the advantages of a continuous-valued risk score when analyzing long-term costs, specifically, (1) it is better able to differentiate high risk groups from low risk groups in terms of their long-term costs; (2) it allows decision-makers to better understand how the average risk level of a “high risk” group targeted for interventions declines as the percent of the population targeted for the intervention increases; and, (3) it allows straightforward calculation of changes in risk score over time and analysis of the relationship between score changes and long-term costs. This latter advantage is particularly important in justifying interventions at midlife to reduce the natural increase in risk factors associated with the aging process.

## METHODS

### Study Sample

The CMS data included Medicare enrollment information and expenditures (referred to as Medicare costs) between 2000 and 2008 for 3849 Framingham Heart Study participants: 740 from the Original Framingham cohort, 2796 from the Second Generation cohort, 61 from the Third Generation cohort, and the rest from special cohorts. Risk scores were calculated from exams done between 1971 and 2007. Midlife CHD risk score (a risk score calculated between age 40 and 50 years which was closest to age 45 years) and sufficient cost data were available only for the Second Generation participants, and so our initial sample was 2796. From this sample, we excluded 672 people who were continuously enrolled in a Medicare managed care program (since cost data were unavailable for them), and those who had not bought into Medicare Part B (since they were likely still working and had other insurance); 67 participants who were under age 65 years but eligible for Medicare because of disability; 15 participants who had only 2 quarters of cost data in the last 6 months of their life; following the approach of Daviglus *et al.* [3], to stabilize cost estimates, 314 participants who did not have at least 8 quarters of cost data; 21 people who had CHD at the time of their midlife risk score and 374 people who did not have a midlife risk score. This left a final sample of 1333 cases. For the analysis using later-life score minus midlife score, which we call “score change”, we excluded an additional 128 people who developed CHD between their midlife score and later-life score. Supplemental Material Table S1 shows details of the exclusions and compares average midlife risk score and average quarterly costs (if available) for the included and excluded cases for each of the criterion.

We required at least 8 quarters of cost data to allow averaging of any post-Medicare eligibility spending “bump” at age 65 years (e.g., because a person may wait until receiving Medicare eligibility to seek treatment for various conditions). Also, for the many people in our data set whose first Medicare costs were after age 65 years (age at time of first costs was 68 years or older for 50% of the participants), we did not have prior enrollment information. These may have been people on a spouse policy or in a Medicare managed care program that changed to fee-for-service because of increased flexibility to obtain various types of treatment. The 8 quarter criterion allowed the costs to be averaged over a long enough period to reduce the impact of any initial eligibility increase in spending. The higher average quarterly costs of those without 8 quarters of data (Supplemental Material Table S1) suggests this type of “bump” does occur.

### CHD Risk Prediction Score

We used the Framingham CHD 10-year risk prediction score [10,11] as a way to combine risk factors into a continuous measure of risk factor burden. The risk score assigns points based on 8 risk factors: sex, age, systolic and diastolic blood pressure (average of 2 readings after the participant has been sitting for 5 min), fasting total and high-density lipoproteins (HDL) cholesterol (based on blood drawn at the examination), self-reported current smoking status (yes/no within the year prior to examination), and diabetes mellitus (random glucose ≥ 200 mg/dL, fasting glucose ≥ 126, or use of insulin or oral diabetes medications). Higher scores denote less healthy profiles. For 3 of the risk factors, negative points are assigned based on particularly healthy levels of the factor. For example, cholesterol <160 mg/dL is assigned −3 points. We included two ages in the analysis: (1) age when risk score was assessed, which is part of the risk score calculation; and (2) age when cost data first became available, which was 2000.

In our main analyses, we used the midlife score, which was the risk score assessed at the exam between age 40 and 50 years that was closest to age 45 years. As a sensitivity analysis, we considered a wider age range, 35–55 years, to define midlife. We also considered a “later-life” score, defined as the risk score assessed closest to but before age 65 years. From the 2 risk scores, we created “score change”, defined as “later-life risk score minus midlife risk score” in order to examine the extent to which people who were more successful in limiting the natural increase in risk score due to aging had lower costs.

### CMS Costs

CMS cost data between year 2000 and 2008 included the following payments: inpatient, institutional outpatient facility (outpatient file), distinct physician office (carrier file), nursing home, home health care, and hospice. We adjusted the cost data for inflation using the Medical Care Consumer Price Index with 2000 as the base year [12]. Also, because costs at the end of life can vary greatly depending upon patient, family, and provider preferences, we dropped from the analysis the last two quarters of participants’ lives, as noted above. For each FHS participant, we aggregated cost data by quarters.

### Statistical Analysis

For descriptive purposes, we reported percentages for binary variables, and mean, standard deviations and percentiles for continuous variables. We ran generalized linear models (GLMs) with a gamma distribution and log link, and used robust standard errors. Based on the results of the Park test, as a sensitivity analysis, we also ran models with a Poisson distribution. The dependent variable was “average quarterly cost”. To reduce the impact of outliers, we top-coded the cost data by replacing any quarterly costs more than 3 standard deviations from the mean with a value equal to 3 standard deviations above the mean. Twenty six cases were top-code to a value of $13,880. As noted above, for all models, we included age at year 2000 as an independent variable. We ran models with the following additional independent variables: Model 1: midlife CHD risk score; Model 2: midlife CHD risk score components—sex, age at midlife exam, average systolic blood pressure, average diastolic blood pressure, total cholesterol, high-density lipoprotein cholesterol, smoking status (yes/no), and diabetes mellitus (yes/no); and Model 3: midlife risk score and “later-life risk score minus midlife risk score”. We converted the coefficient for each variable into the percentage change in expected quarterly costs per one unit increase in the variable. For continuous variables, one unit corresponds to one standard deviation (SD); for binary variables, one unit equals one. The percentage change is calculated as exp(original parameter estimate on log scale × one unit) − 1. As a summary statistic of model performance, we reported the correlation between predicted and actual quarterly costs and a 95% confidence interval, which was estimated from 600 bootstrap replications.

We conducted our analyses using Stata Statistical Software: Release 13, StataCorp, College Station, Texas. A variable was considered statistically significant if *p* < 0.05 in a two-tailed test. Analyses were done between 2013 and 2016. The Framingham Heart Study was reviewed by the Boston University Medical Campus Institutional Review Board (IRB) and all participants signed informed consents. The study reported here, which did not require additional informed consents, was approved by the Boston University Charles River Campus IRB, protocol number 3171E on April 29, 2013.

## RESULTS

For the main exclusion reasons, average midlife risk score was not statistically significantly different for those in fee-for-service compared to those in managed care or without Medicare Part B (672 participants); or for those with at least 8 quarters of cost data compared to those without 8 quarters of data (314 participants), though for the latter comparison, average quarterly costs were significantly higher for those with less data (see Supplemental Material, Table S1). The midlife score was higher for those not meeting the age eligibility criterion (67 participants), for those with quarterly costs only in the last 6 months of life (15 participants), and for those with CHD at midlife assessment (21 participants). For those without a midlife risk score (374 participants), their first-time risk score (which sometimes was earlier than midlife and sometimes later) was noticeably and statistically significantly higher than those with a midlife score, as were their average quarterly costs. Overall, those in our final sample were a somewhat healthier subset of our initial sample. Table 1 shows the average values for the risk score components.

We reported the descriptive statistics on risk scores calculated from the components, as well as costs and the time participants were followed in Table 2. For the main cohort of 1333, the average (standard deviation, SD) midlife risk score was 2.77 (3.64) and the distribution was fairly symmetrical (see Supplemental Material). Average Medicare quarterly costs had a strong right skew (see Supplemental Material), a shape consistent with our use of the gamma distribution in the GLMs. For 50% of the sample, there was a 25-year or more interval between their midlife score and when the first quarter cost data were available; and for 75% there was at least a 20-year period. Those who survived to assessment of later-life risk score without developing CHD had somewhat lower average midlife score, 2.50. Most of the difference between later-life score and midlife score was due to the older age of the later-life group (about 5 of the 6 points difference for a person with a median score). The correlation between the midlife risk score and the later-life score was 0.47. Similar to the midlife risk score, average costs for the smaller cohort who had a usable later-life score were slightly lower.

Table 3 shows the results of the three different models. All of the models include the variable “age at year 2000”. As indicated above, our Medicare cost data were for the years 2000 to 2008. As shown in Table 2, the average age of study participants in 2000 was 66.7 (SD = 5.2) but it varied from a low of 57 years to a high of 77 years. Thus, the up to 9 years of cost data corresponded to different chronological ages, depending on age at 2000. The coefficient for this variable in Model 1 was 21.8. This means that for 2 individuals whose age at year 2000 differs by 5.2 years (the SD of the variable), the quarterly costs of the older person are predicted to be 21.8% higher than the quarterly costs of the younger person. Model 1 also includes midlife risk score, which had a coefficient of 26.2. This means that a one SD increase in midlife score (which corresponds to 3.64, Table 2) was associated with a 26.2% increase in average quarterly costs. Using the results of Model 1, the predicted average quarterly cost for an individual with mean age in 2000 (66.7 years) and with a CHD risk score at the 75th percentile was $2795, 38% more expensive than someone with a CHD risk score at the 25th percentile (which is 5 points lower).

Model 2 shows results when the risk factors that comprise the CHD score were included as separate variables. Interpretation of coefficients associated with the continuous variables (*i.e*., the percentage change in quarterly costs for a one SD change in the variable) is the same as in Model 1. For the binary variables, the coefficient is the percentage increase in quarterly costs when the value of the variable changes from 0 to 1. Only 4 of the 8 risk score component variables were statistically significant: age, HDL cholesterol level, current smoking and diabetes. The impact of diabetes, which has a coefficient of 109.8, indicates that compared to somebody without diabetes, someone with diabetes has quarterly costs that are almost 110% higher. The correlation between predicted and actual costs was slightly higher than when the risk score components were used as the independent variables, but the 95% confidence intervals for the correlations were wide and the differences were not statistically significant.

The results from Model 3 show that, adjusting for midlife risk score, a one SD increase in change score (3.86 points) was associated with a 12.2% increase in average quarterly costs. Someone of average age at 2000 with an average midlife risk score and whose change score was at the 75th percentile had predicted quarterly costs of $2431, 20% more expensive than someone whose change score was at the 25th percentile (which was 6 points lower).

We estimated the average quarterly cost and average midlife risk scores for cases in different percentiles of the distribution of predicted costs from the model with age at 2000 and midlife risk score (*i.e*., Model 1), as shown in Table 4. The average quarterly costs for someone whose predicted average quarterly costs were in the top 5% were $4378, 1.73 times higher than the overall average quarterly cost for the entire sample ($2535); average quarterly costs for someone in the top 20% were 1.5 times higher than overall average costs. Average quarterly costs for those in the lower risk group (bottom 20% in terms of predicted costs) were 57% of overall average costs and 51% of those not in the lower risk group. Of note, for studies of healthy aging, those in the lowest 20% in terms of risk have very similar long-term costs and thus might be the relevant group to analyze.

Table 5 shows the average values of the risk score components for those at high risk (defined as the top 20% of predicted costs, using Model 1), those at low risk (defined as the bottom 20% of predicted costs), and the middle group at medium risk, those between the 20th and 80th percentile. As seen in the Table, risk factors become less favorable for those in increasingly higher risk groups.

In sensitivity analyses, when we expanded the definition of mid-life from age 40–50 to age 35–55 years, the eligible cohort increased from 1333 to 1472. The expanded cohort was somewhat less healthy, with a midlife risk score of 3.19 (*vs*. 2.77 in the initial cohort) and higher average quarterly costs ($2634 *vs*. $2535). Using Model 1, the increase in average quarterly costs associated with a one unit increase in midlife score (3.84 points) was 28.2% (compared to 26.2% when using the initial sample). However, almost all of the increase (1.6% of the 2.0%) is because “one unit” (the SD) is larger. Using the Poisson distribution instead of the gamma distribution had no substantive impact on results.

## DISCUSSION

We show that the FHS CHD 10-year risk score in mid-life differentiates groups with substantially different health care costs occurring, on average, almost 24 years after risk assessment. Also, adjusting for midlife risk score, a smaller change score between midlife and later-life was associated with lower future costs. When we reweighted the Framingham risk score components, only 4 of the variables had a statistically significant relationship with costs: age, HDL cholesterol, smoking status, and diabetes. The key role of diabetes in predicting future costs is noteworthy given the current obesity epidemic and the rise in the incidence of diabetes.

Schauffler *et al.* [6], based on a sample of 1053 elderly (age 63 to 93) FHS participants, developed a model predicting Medicare reimbursements per person over 2 years (1984 and 1985) based on the 2-year predicted probability of cardiovascular disease at an exam given in 1982 or 1983. The regression model also controlled for other risk factors and prior health services use. They found that the average level of risk was associated with 19% higher Medicare costs compared to someone with no elevated risk. Interestingly, our model predicts that costs occurring on average 23 years after an average midlife exam risk score (2.77) are 19% higher costs than if the risk score had been zero (equivalent to no elevated risk). It is worth noting that “no elevated risk” in our case is not the healthiest group, which has a risk score of −8, but the 25th percentile of the risk score distribution (Table 2).

Valero-Elizondo *et al.* [7] studied 15,651 participants at age 40 years or older in the 2012 Medical Expenditure Panel Survey. Overall, 37.8% of the sample had an optimal cardiovascular risk factor profile and 17.4% had a poor profile. Among participants without CVD, annual expenditures were $4031 by those with a poor profile compared to $2560 by those with an optimal profile, or 1.6 times higher. In our sample, the 20% with the highest midlife risk score had average quarterly costs 23 years later that were 2.1 times higher than the 40% with the lowest midlife risk score. Aaron *et al.* [8] analyzed Medicare expenditures in the year after a baseline visit between 2003 and 2007 for 6262 participants in the Reasons for Geographic and Racial Differences in Stroke study. Overall, 17.2% of the sample had 0–1 ideal risk factors (high risk group) and 6.4% had 5–7 ideal risk factors (low risk group). Those in the highest risk group had annual costs of $9147, 2.2 times higher than those in the lowest group. In our sample, the 20% in the highest group had quarterly costs that were 2.7 times higher than the 10% in the lowest group.

As noted, there have been two studies examining future costs occurring many years after CVD risk assessment. Daviglus *et al.* (1998)[3] analyzed 7039 men and 6757 women from the Chicago Heart Association Detection Project in Industry who were 40 to 64 years old between 1967 and 1973 and who survived to have at least two years of Medicare coverage between 1984 and 1994. Based on the presence of 6 CVD risk factors coded as present or not, they found a low risk group (4.2% of the population) whose costs were 57% of those not at low risk (averaging results for men and women). We were able to identify a much larger low risk group (20% of the sample) whose average quarterly costs were 54% of those not at low risk. Allen *et al.* (2017)[5] expanded and extended follow-up of the cohort initially considered by Daviglus *et al.* Over 25,000 study participants were placed into one of four risk categories (% of participants in each category are shown in parentheses): favorable levels on all risk factors (6% of the sample), one or more elevated risk factors (19%), 1 high risk factor (40%) and 2 or more high risk factors (35%). Median annual costs for those in the highest risk group were about $5500 higher than those in the lowest risk group. The difference in average quarterly costs of someone in the 10% of our sample with lowest risk compared to someone in the 40% of the sample with highest risk was $1936 (=$3343 − $1407, Table 4), or $7744 annually, about 40% higher than the difference reported by Allen *et al.*, Willis *et al.* (2015)[4] using data on 4906 participants from the Cooper Center Longitudinal Study, examined the relationship between midlife CVD risk profile (average age 56 years) and Medicare costs. They defined poor, intermediate, and ideal cardiovascular health based on 7 risk factors. The 31% of the population with an unfavorable risk profile (0–2 ideal values for risk factors) had 37% higher costs than the 18% of the sample with a favorable cardiovascular profile (5–7 ideal values for risk factors). In our sample, the spread between the high and low risk groups was much larger: those in the top 30% in terms of risk had 2.4 times higher average quarterly costs than the 20% of the sample at lowest risk. Thus, compared to a number of studies that have defined risk categories based on a small number of risk categories, the Framingham continuously-valued risk score was able to identify comparable high and low risk groups in terms of the percentage of the sample in the group but with larger differences in costs between the two groups.

For organizations that manage population health, a continuous-valued risk score allows flexibility in identifying risk groups (e.g., see Table 4) and modifying the intensity of interventions based upon level of risk. For example, planners might target those in the top 5% in terms of risk, who have projected costs over 70% of average, with particularly aggressive personalized interventions; those with risks in the next 15% of the population with somewhat less intensive personalized interventions; and the next 30% with less-personalized population-based interventions. The bottom 40% of the population is at relatively low risk and might not be the focus of prevention efforts.

Perhaps the most important value of a continuous risk score is that it allows straightforward calculation of a change score and thus facilitates analysis of the longer-term cost implications of efforts to improve risk factor profiles. Because the Framingham risk score increases as a function of age, a later-life score will usually be higher than a mid-life score. However, an intervention that is able to reduce, for a person with a median midlife score, the change score of from the 75th percentile to the 25th percentile would result in almost a 20% reduction in longer-term quarterly costs. In contrast, an intervention that is able to reduce a person’s midlife score from the 75th percentile to the 25th percentile would result in a 38% reduction in quarterly costs. This type of comparison provides some sense of the relative longer-term cost advantages of interventions to reduce midlife risk score compared to interventions to reduce increases in risk score from midlife to later-life. Though later-life interventions have less potential impact, there are still substantial economic benefits from limiting the increase in the risk prediction score.

Nasir *et al.* [13] in an editorial accompanying the Allen *et al.* [5] study raised the difficult question “who should shoulder the responsibility” to support prevention efforts that may not have economic payoff until many years later. Workplace interventions have some potential [14]. Studies have shown there are also differences in current health care expenditures in younger populations between those with favorable and unfavorable CVD risk profiles. For example, in the Valero-Elizondo *et al.* [7] study discussed above, almost 75% of the sample was under age 65. Osundu *et al.* [15], who studied a relatively young employed population, found lower health care expenditures between those with optimal CVD risk profiles compared to those with moderate and poor profiles ($4282, $5824 and $10,104 respectively). So, there are also short-term economic incentives to encourage the private sector to implement programs to improve employee risk profiles. CMS obviously has a strong incentive to support primary prevention of CVD risk factors, as well as efforts to limit further increase in risk of those already at moderate or high risk at midlife. Also, the number of people joining Medicare managed care programs continues to increase. Since many of these plans are offered by the same large insurers that are active in private insurance markets for the under 65, incentives are increasing for private insurers to more actively focus on risk factor reductions in younger populations. All of this is positive because of the large economic costs associated with the failure to control CVD risk factors.

The challenge is how to design programs to most effectively impact risk behaviors. In a recent randomized controlled trial of a workplace wellness program, Song and Baicker [16] found that though there were improvements in some health reported behaviors among the study group, there were no significant differences in clinical measures of health or health care spending after 18 months. As Abraham noted in an accompanying editorial [17], randomized trials of wellness programs, which avoid the healthy volunteer bias of observational trials (*i.e*., participants in the trial are more motivated to change their behaviors), have not found significant impacts, similar to findings from rigorously designed nonrandomized studies. He went on to note the need to “innovate, test and evaluate novel designs” (p 1463) and suggested the potential value of more targeted approaches that focus on those individuals with elevated risk. As illustrated above, a continuous-valued risk score like the Framingham risk score facilitates identifying these particularly high-risk groups. One increasingly used approach to encourage behavior change is to offer financial incentives. However, as implied by the title of a recent JAMA Viewpoint article by Thirumurthy *et al.* [18]—“The uncertain effect of financial incentives to improve health behaviors”—financial incentives are unlikely to be the panacea, though more carefully designed and implemented programs may have potential. Needless to say, one of the great challenges facing our health care system is how to increase healthy behaviors in the population.

### Limitations

There are limitations to our study. Though throughout the paper the discussion focuses on cardiovascular risk factors, we used the Framingham Coronary Heart Disease 10-year risk score. The reason for this is that the Framingham database available to us did not contain certain variables necessary to calculate the Framingham CVD risk score (in particular, certain clinical variables necessary to identify the presence of diabetes). Nonetheless, the risk factors in both scores are largely similar. If anything, it is likely a continuous-valued CVD risk score would have provided even better risk group differentiation than that found using the CHD score.

The sample size in our study was relatively small and included mostly whites from a largely middle class community in New England. Also, we examined a relatively healthy sample at both midlife, as shown by the lower midlife risk score for most of the inclusion criteria, and at later-life, since the latter group excluded individuals that developed CHD between their midlife and later-life score. Although midlife scores were similar for those in fee-for-service Medicare and those not, their resource utilization patterns were not necessarily similar. The generalizability of results to those not in Medicare fee for service is thus uncertain. Finally, like all observational studies, we cannot rule out residual confounding and have not established a causal relationship between risk scores and costs.

## CONCLUSIONS

Our study confirmed the longer-term Medicare costs of unfavorable CVD risk factor levels at midlife. However, it also illustrated the advantages of a continuous-valued risk score as opposed to categorizing people into a small number of categories based on the presence of combinations of risk factors. With the Framingham score, we were able to better differentiate high and low risk groups with larger cost differences; determine the higher costs associated with increasing risk, thus providing a basis for more appropriately targeting interventions of different intensity for those in different risk groups; and, most important, associate a risk change score with longer-term costs and thus demonstrate the potential cost savings of interventions that limited the increase in risk score between midlife and later-life.

## Supplementary Material

supplemetal

## Figures and Tables

**Table 1.** Means (standard deviations) and percentages for risk score components for all cases.

Framingham CHD Risk Score Components	All Cases (*n* = 1333)
Sex (% women)	55.5
Age at midlife exam, years	45.4 (2.3)
Systolic blood pressure, mm Hg	122 (15)
Diastolic blood pressure, mm Hg	80 (9)
Total cholesterol, mg/dL	210 (36)
HDL cholesterol, mg/dL	53 (17)
Smoker (% yes)	34.2
Diabetes (% yes)	1.9

HDL, high density lipoprotein.

**Table 2.** Summary statistics for key variables for all cases.

Participant Characteristics	Mean (SD)	Percentiles				
		Min *	25th	Median	75th	Max
**Total sample, *n* = 1333**						
Midlife CHD risk score	2.77 (3.64)	−8	0	3	5	19
Average quarterly costs $	2535 (2856)	0	679	1501	3347	13,880
Number years from midlife score to first quarter of costs	23.49 (3.89)	15	20	25	27	34
Number of quarters of cost data	26.79 (9.46)	8	19	29	36	36
Age in 2000, years	66.7 (5.2)	57	62	67	71	77
**Sample with later-life score, *n* = 1205**						
Midlife CHD risk score	2.50 (3.59)	−8	0	3	5	14
Later-life CHD risk score	8.67 (3.80)	−8	6	9	11	21
Score change: Later-life score - Midlife score	6.17 (3.86)	−6	3	6	9	21
Average quarterly costs $	2344 (2687)	0	636	1413	3123	13,880
Age in 2000, years	66.5 (5.2)	57	62	66	71	77

* As indicated in the text, the scoring algorithm assigns negative points for particularly healthy levels of certain variables. SD, standard deviation; CHD, coronary heart disease.

**Table 3.** Percentage Change in Quarterly Costs for One Unit Increase in Independent Variables for Different Models *.

Independent Variables	Model 1		Model 2		Model 3	
	Estimate	*P*-Value	Estimate	*P*-Value	Estimate	*P*-Value
Age at Year 2000	21.8 (14.9, 29.0)	0.000	23.4 (15.5, 31.8)	0.000	19.7 (12.6, 27.3)	0.00
Midlife CHD Score	26.2 (18.4, 34.5)	0.000			29.8 (19.8, 40.6)	0.00
Sex: Woman			−2.6 (−14.8, 11.4)	0.70		
Age at Midlife Exam			2.0 (−4.8, 9.3)	0.58		
Average Systolic Blood Pressure			4.8 (−6.0, 16.8)	0.40		
Average Diastolic Blood Pressure			6.4 (−3.9, 17.9)	0.22		
Total Cholesterol			1.5 (−4.8, 8.1)	0.61		
HDL Cholesterol			−11.2 (−17.7, −4.3)	0.003		
Current Smoker: Yes			22.5 (7.8, 39.2)	0.002		
Diabetes Mellitus: Yes			108.7 (42.3, 206.0)	0.000		
Late-life Score-Midlife Score					12.2 (4.5, 20.4)	0.001
Number of cases	1333		1333		1205	
Correlation †	0.264		0.283		0.271	
95% Confidence Interval	0.22–0.31		0.23–0.34		0.21–0.33	

* Estimates are on a scale that shows the percentage change in expected quarterly costs for a one unit increase in the independent variable. A one unit increase equals one standard deviation for continuous variables and one for binary variables (*i.e*., if the binary variable changes from no to yes).

†: Correlation between predicted cost and actual cost. Adjustments were made as follows: Model 1, age in 2000, midlife CHD risk score; Model 2, age in 2000, midlife CHD risk score components–sex, age at midlife exam, average systolic blood pressure, average diastolic blood pressure, total cholesterol, high-density lipoprotein cholesterol, smoking status (yes/no), and diabetes mellitus (yes/no); and Model 3, age in 2000, midlife CHD risk score, and “later-life risk score minus midlife risk score”. CHD, coronary heart disease; HDL, high-density lipoprotein.

**Table 4.** Average quarterly costs for different subgroups defined by their predicted costs *.

Percent of Cases	Average Quarterly Cost $	Ratio of Average Quarterly Costs to Overall Average †	Average Midlife CHD Risk Score
Top 5	4378	1.73	8.01
Top 10	3837	1.51	7.08
Top 20	3753	1.48	6.33
Top 30	3455	1.36	5.81
Top 40	3343	1.32	5.40
Top 50	3110	1.23	4.94
Bottom 50	1945	0.77	0.55
Bottom 40	1788	0.71	0.04
Bottom 30	1621	0.64	−0.78
Bottom 20	1445	0.57	−1.73
Bottom 10	1407	0.56	−2.99
Bottom 5	1401	0.55	−3.71

* Using the GLM with age at 2000 and midlife CHD risk score as independent variables.

†: Overall average quarterly cost = $2535. CHD = coronary heart disease.

**Table 5.** Means (standard deviations) and percentages for risk score and risk score components by risk category *.

Framingham CHD Risk Score and Risk Score Components	Low Risk (*n* = 267)	Medium Risk (*n* = 800)	High Risk (*n* = 266)
CHD risk score	−1.73	2.75	6.33
Sex (% women)	74.9	54.4	39.5
Age at midlife exam, years	44.9 (1.9)	45.3 (2.3)	46.4 (2.4)
Systolic blood pressure, mm Hg	114 (11)	122 (13)	132 (16)
Diastolic blood pressure, mm Hg	74 (8)	80 (8)	86 (10)
Total cholesterol, mg/dL	200 (33)	210 (36)	218 (36)
HDL cholesterol, mg/dL	64 (16)	53 (16)	42 (12)
Smoker (% yes)	12.7	34.3	55.6
Diabetes (% yes)	0	0	9.4

* Risk categories are defined based on cost predictions using the age at 2000 and midlife risk score as independent variables. Low risk cases are in bottom 20% in terms of risk predictions, and high risk are in top 20% and medium risk are those between the 20th and 80th percentile. HDL, high density lipoprotein.

## ABBREVIATIONS

CHD: Coronary heart disease
CMS: Centers for Medicare and Medicaid Services
CVD: Cardiovascular disease
FHS: Framingham Heart Study
GLMs: Generalized linear models
HDL: High-density lipoproteins
IRB: Institutional Review Board
SD: Standard deviation
