# Supplementary material for: Advantages of Continuous-Valued Risk Scores for Predicting Long-Term Costs: The Framingham Coronary Heart Disease 10-Year Risk Score
Source: Adv Geriatr Med Res. Author manuscript; Available in PMC 2019 Aug 23. (PMC6707532; doi:10.20900/agmr20190004)
Supplement: supplemetal [file NIHMS1039940-supplement-supplemetal.pdf]

SUPPLEMENTAL MATERIAL

Table S1. Number of cases excluded for each criterion and risk score and cost comparisons.

Inclusion Criteria	Number of Cases	Number with Midlife Risk Score	Average Midlife Risk Score	Number with Quarterly Costs	Average Quarterly Cost $
**Initial Sample**	2796				
**1. Fee for service**					
Yes	2124	1666	2.91	-	-
No	672	565	2.88	-	-
**2. Age eligibility** *					
Yes	2057	1609	2.86	2042	3031
No	67	57	4.14 #	66	4051
**3. Quarterly costs**					
Yes	2042	1601	2.84	-	-
No †	15	8	6.88 **	-	-
**4. ≥8 quarters costs**					
Yes	1728	1346	2.80	1728	2810
No	314	255	3.08	314	4249 **
**5. No CHD at exam**					
Yes	1707	1333	2.77	1707	2796
No	21	13	5.62 **	21	3913
**6. Midlife risk score**					
Yes	1333	1333	2.77	1333	2686
No	374	366 ‡	4.11 §,**	374	3188 *
**Final Sample**	1333				
**Final Change Score Sample** ||	1205				

*: Enrollment in Medicare is based on age ≥ 65 and is not based on having a disability. †: We exclude the last 2 quarters of cost data prior to death. Thus, those in fee for service without quarterly costs incurred all costs in the last 2 quarters before death. ‡: These are people who have a risk score but none of their risk scores are in the midlife range. §: This is the first risk score for those without a midlife score. Sometimes the first score is from cases below age 40 and sometimes from cases above age 50. The average age of the midlife score for the 1333 cases is 45.4 (SD = 2.31); the average age of the first risk score for the 374 cases is 48.7 (SD = 8.3), which in part accounts for the higher score. ||: For the change score analysis, 128 cases that developed CHD between the midlife exam and the later-life exam were excluded. #: $0.01 < p < 0.05$; **: $p < 0.01$. CHD, coronary heart disease.

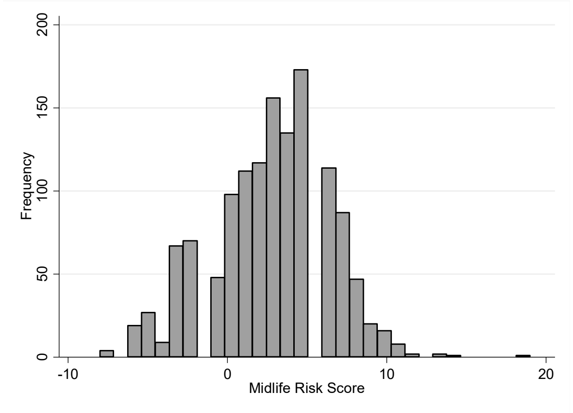

Figure S1. Distribution of Midlife Framingham Risk Scores.

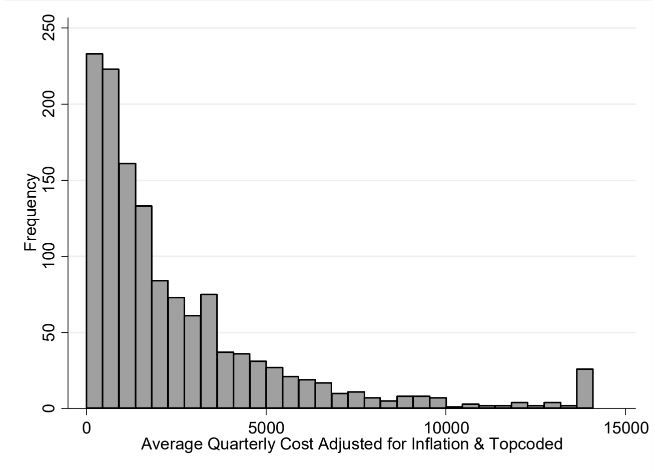

Figure S2. Distribution of Average Medicare Quarterly Costs.
